# Microstrip Antenna Bandwidth Optimization for RF Microsystems Using Swarm Intelligence and Reinforcement Learning

**DOI:** 10.3390/mi17060680

**Published:** 2026-05-30

**Authors:** Shaolong Cao, Yu Shao, Jie Zhang, Yang Wang, Ju Tan, Kai Zhu, Lianghong Li

**Affiliations:** 1 School of Communications and Information Engineering, Chongqing University of Posts and Telecommunications, Chongqing 400065, China; d200101001@stu.cqupt.edu.cn (S.C.); jie.zhang@ranplanwireless.com (J.Z.); wangyang@cqupt.edu.cn (Y.W.); j5802397@gmail.com (J.T.); s210101216@stu.cqupt.edu.cn (K.Z.); leelianghong@outlook.com (L.L.); 2 Ranplan Wireless Network Design Ltd., Cambridge CB23 3UY, UK

**Keywords:** microstrip antenna, bandwidth extension, crayfish optimization algorithm, surrogate model, reinforcement learning

## Abstract

As essential radiating elements in RF and microwave microsystems, microstrip antennas require sufficient bandwidth to ensure stable operation, integration flexibility, and overall microsystem performance. From a microsystem optimization perspective, this paper proposes a bandwidth extension method for microstrip antennas that combines swarm intelligence and reinforcement learning. The proposed ICOA-TD3 framework is designed to enhance antenna bandwidth within target frequency bands and thus improve the performance robustness of compact RF microsystems. In the proposed method, an improved crayfish optimization algorithm (ICOA) is first used to explore the global design space and achieve global bandwidth enhancement, followed by the Twin Delayed Deep Deterministic Policy Gradient (TD3) algorithm for local refinement and further exploitation of the antenna structure’s bandwidth potential. In Experiment 1, the impedance bandwidth ($S_{11}\leq -10\;\mathrm{dB}$) is increased by up to 200%. In Experiment 2, the impedance bandwidth ($S_{11}\leq -10\;\mathrm{dB}$) and axial-ratio (AR) bandwidth (AR≤3dB) are improved by up to 27% and 250%, respectively. The results indicate that the proposed method is a feasible solution for bandwidth-oriented optimization of microstrip antennas and is promising for the intelligent design of high-performance RF microsystems.

## 1. Introduction

Antenna bandwidth optimization plays a crucial role in antenna performance optimization, and bandwidth enhancement is a key research focus within this area. This is because antenna bandwidth is one of the most important indicators of antenna performance, reflecting the antenna’s ability to effectively radiate or receive the majority of power across a given frequency band. Previously, research on antenna bandwidth enhancement—for example, increasing the impedance bandwidth ($S_{11}\leq -10\;\mathrm{dB}$)—often relied heavily on the designer’s personal experience and involved extensive, time-consuming tuning and repeated adjustments, making this process exceptionally difficult and tedious. To alleviate these difficulties, researchers have begun to introduce swarm intelligence algorithms, reinforcement learning, surrogate models, and hybrid schemes that integrate multiple algorithms to improve the efficiency of antenna bandwidth enhancement.

In the existing literature, numerous methods for antenna bandwidth enhancement have been proposed. Reference [1] proposes a feature-based framework for antenna performance optimization, in which characteristic points are extracted from full-wave electromagnetic simulations and used to optimize the antenna. Reference [2] presents an antenna bandwidth-optimization approach that combines neural networks with population-based algorithms, where a convolutional neural network is employed to construct a surrogate model and particle swarm optimization is used to search for designs with wider bandwidth. Reference [3] proposes a machine-learning-based framework for antenna shape optimization that integrates optimization algorithms with self-supervised learning strategies to optimize antenna geometry. Reference [4] proposes an efficient machine learning framework for multi-objective antenna design that optimizes the antenna by learning the relevant features of multiple objectives using a hybrid neural network architecture. Reference [5] proposes a framework for fast antenna bandwidth optimization that accelerates the process by adopting an augmented tree-structured Bayesian estimation algorithm. Reference [6] proposes a framework for balancing antenna gain maximization and bandwidth enhancement, which employs sequential constrained optimization to achieve a balance between these objectives. Among these frameworks, some rely on a single algorithm, whereas others combine multiple techniques to construct an antenna optimization framework; however, their bandwidth-optimization performance remains limited.

Swarm intelligence algorithms have been widely used in the global optimization of antenna bandwidth. In reference [7], an improved discrete-mutated particle swarm optimization (PSO) method for designing leaky-wave antennas with low sidelobes and enhanced bandwidth is proposed. It improves bandwidth-optimization performance by introducing a non-inertial weight function, an asymmetric learning-factor function, and a Gaussian-perturbed particle-mutation operator into the particle swarm algorithm. In reference [8], a trust-region parallel Bayesian optimization framework for antenna bandwidth enhancement is proposed. It accelerates antenna optimization by introducing a multi-point acquisition function and a trust-region-based design space into the Bayesian optimization process. In reference [9], a fast non-dominated sorting genetic algorithm (NSGA-II)-based method for joint optimization of antenna bandwidth and reflection coefficient is proposed. It obtains the optimal combination of antenna parameters that yields the maximum bandwidth and a low reflection coefficient at the center resonant frequency using a design of experiments (DoE) combined with NSGA-II. In reference [10], a hybrid optimization algorithm that combines the mutation and crossover strategies of the differential evolution (DE) algorithm with the observer-bee phase of the artificial bee colony (ABC) algorithm is proposed to improve the bandwidth of rectangular microstrip antennas. In reference [11], a dynamic hybrid binary particle swarm optimization (BPSO) algorithm is proposed to improve the bandwidth of inverted-F antennas. Reference [12] proposes a hybrid method that combines the gray wolf optimizer (GWO) with an artificial neural network (ANN) to achieve comprehensive optimization of in-band gain flatness, impedance matching, and filtering characteristics. Reference [13] proposes an antenna bandwidth prediction algorithm that combines a swarm-intelligence-based optimization algorithm with an ANN, which is built on a guided whale optimization algorithm enhanced by adaptive PSO to tune the parameters of a long short-term memory (LSTM) deep neural network (DNN), thereby achieving high-precision antenna bandwidth prediction. However, these swarm intelligence algorithms still suffer from either limited convergence accuracy or slow convergence speed.

Applying a surrogate model to replace electromagnetic simulation in antenna optimization can significantly reduce computational cost and optimization time. Current research has increasingly focused on improving the predictive performance of surrogate models. Reference [14] proposes a performance-driven surrogate modeling strategy, which achieves substantially better prediction accuracy than nested Kriging methods without increasing the size of the training dataset. Reference [15] proposes a method to reduce the cost of building surrogate models within a nested Kriging framework, leveraging inverse-sensitivity analysis, iterative correction, and feature-based accelerated gradient search to lower the overall modeling cost. Reference [16] proposes an adaptive Bayesian neural-network surrogate–assisted differential evolution antenna optimization scheme, which enhances antenna gain bandwidth and stabilizes sidelobe levels in the millimeter-wave band. However, despite these advances, the above surrogate models still suffer from limited prediction accuracy.

Machine learning has been widely used in antenna optimization. Reference [17] proposes a machine learning method based on a multilayer perceptron (MLP) artificial neural network to optimize the performance of printed inverted-F antennas. Reference [18] proposes a general machine-learning-based framework for optimizing antenna performance. Reference [19] proposes a deep-learning approach to analyze the performance of dual-polarized high-isolation antennas. Reference [20] proposes an artificial-intelligence-based method for searching antenna designs with the widest achievable bandwidth. Reference [21] proposes a machine learning method based on data augmentation and random forests to optimize the AR bandwidth of circularly polarized omnidirectional base-station antennas. Reference [22] proposes a machine learning method that incorporates domain expertise to optimize antenna bandwidth. Reference [23] proposes a reinforcement-learning-based image-mapping topology-optimization scheme to optimize antenna structures. However, these machine learning methods either require large training datasets or exhibit limited convergence and optimization accuracy.

Current methods for extending antenna bandwidth, including those mentioned in the aforementioned literature, suffer from limitations such as limited extension effects, time-consuming iterative convergence processes in global algorithms searching for global optima, low prediction accuracy of surrogate models, and limited exploration capabilities of local algorithms in finding local optima. This paper addresses these issues by proposing a framework for extending the bandwidth of microstrip antennas, providing a feasible solution. The main innovations of this paper are as follows.

(1) A bandwidth extension method for microstrip antennas, named ICOA-TD3, is proposed. The proposed method performs extension based on the antenna’s original frequency band, which can ensure that the extended frequency band contains the original operating frequencies of the antenna, so that the antenna will not fail to operate within the original band after bandwidth extension. Meanwhile, it can provide a sufficient margin to account for tolerances that may arise during the subsequent manufacturing process of the antenna. The overall method is composed of a swarm intelligence algorithm, a surrogate model, and reinforcement learning, and its extension process consists of two stages: in the first stage, the swarm intelligence algorithm is co-simulated with CST Studio Suite 2022 software to search for the global optimal solution, so as to achieve global bandwidth extension; in the second stage, bandwidth extension is further carried out on the basis of the frequency band obtained in the first stage, in which the reinforcement learning algorithm is co-simulated with the surrogate model to explore the local optimal solution, so as to achieve local bandwidth extension.
(2) An ICOA is proposed. This ICOA significantly reduces the algorithm’s running time without affecting its performance. The ICOA moves the call to the fitness value obtained from the CST software simulation from within the original algorithm’s loop body to outside the loop body. This means that each time the loop search begins, only one CST software call is needed, saving a significant number of calls compared to the original algorithm.

## 2. Introduction to the Method Framework

### 2.1. Method Overview

**Figure 1:**
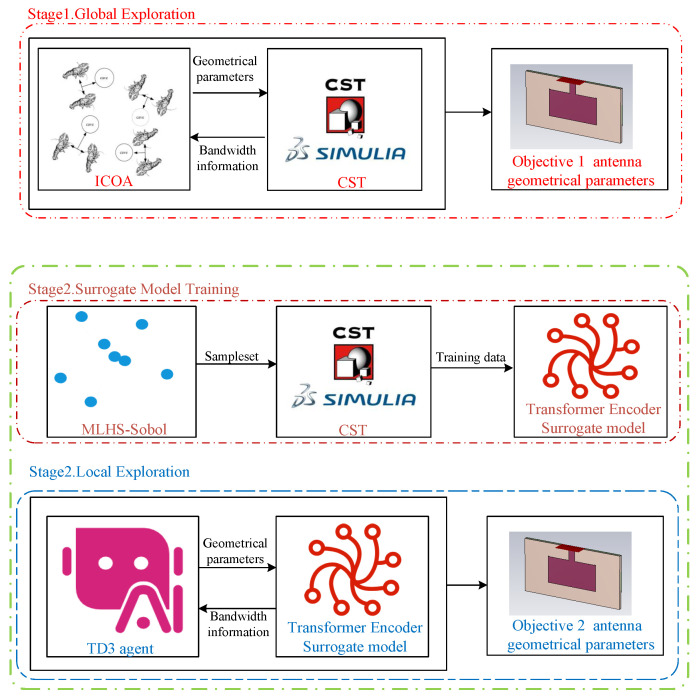
Framework of the proposed method.

This study focuses on the impedance bandwidth ($S_{11}\leq -10\;\mathrm{dB}$) and the AR bandwidth, with the objective of extending these two types of bandwidth for microstrip antennas. The framework of the proposed method is illustrated in Figure 1. The overall framework consists of two parts: the global search part and the surrogate-model construction and local search part.

The first part performs a global search using co-simulation between the ICOA and CST. Specifically, the microstrip antenna geometric parameter data generated by ICOA are fed into CST for simulation to obtain the bandwidth-amplitude information. Based on this information, ICOA iteratively updates the geometric parameter data of the microstrip antenna to obtain the global optimum, thereby achieving the objective of this stage.

In the second part, the surrogate-model construction module adopts the MLHS-Sobol sampling method [24], which combines Maximin Latin Hypercube Sampling (MLHS) with Sobol sampling, to collect samples of the microstrip antenna geometric parameter data. These samples are then fed into CST for simulation to obtain the corresponding bandwidth-amplitude data. The geometric parameter samples together with the bandwidth-amplitude data jointly constitute the training dataset, which is used to train a surrogate model built upon the Encoder module of the Transformer architecture, thereby yielding the Transformer-based surrogate model. The local search module of the second part adopts the TD3 reinforcement learning optimization algorithm [25] and co-simulates it with the Transformer surrogate model to perform the local search. Specifically, the microstrip antenna geometric parameter data generated by the TD3 algorithm are fed into the Transformer surrogate model for simulation to obtain the predicted bandwidth-amplitude information. Based on this information, the TD3 algorithm iteratively updates the geometric parameter data of the microstrip antenna to obtain the local optimum, thereby achieving the objective of this stage.

### 2.2. Improve the Crayfish Optimization Algorithm

To achieve the research objective of Phase 1, we adopt the Crayfish Optimization Algorithm (COA) [26], one of the swarm-intelligence optimization algorithms. In our previously published work [27], a comparison among several swarm-intelligence optimization algorithms showed that COA exhibits clear advantages in both convergence speed and convergence accuracy; that is, the algorithm achieves a higher speed and higher accuracy when searching for the optimal solution. We therefore select it as the optimization algorithm for the objective of Phase 1.

During the experiments, we found that COA requires frequent invocations of the CST software to obtain the bandwidth-amplitude values, thereby consuming a considerable amount of time. To reduce such frequent invocations, we improve the algorithm so as to enhance the simulation efficiency. Specifically, in Formula (11) of the original work, reproduced as Formula (1) below, the part that computes the fitness value of the food position (i.e., ${\mathrm{fitness}}_{\mathrm{food}}$) needs to be invoked by the algorithm at every iteration to obtain the corresponding fitness value.

(1) $$Q=C_{3}\;\times \mathrm{rand}\;\times \left(\frac{{\mathrm{fitness}}_{i}}{{\mathrm{fitness}}_{\mathrm{food}}}\right)\;$$

In the above formula, *Q* denotes the size of the food, $C_{3}$ is the food factor, ${\mathrm{fitness}}_{i}$ denotes the fitness value of the *i*-th crayfish, and ${\mathrm{fitness}}_{\mathrm{food}}$ denotes the fitness value of the food position. In the original work, since the fitness function is constructed from a mathematical formula, the calculation can be completed rapidly. In our study, however, since this fitness value must be obtained by invoking CST simulation, a large amount of simulation time is consumed at every iteration. After a combined analysis of the original work and the program code it provides, we found that this part can be moved entirely outside the iterative update loop. This is because, when calculating the ${\mathrm{fitness}}_{\mathrm{food}}$ value, the variable food is generated outside the loop body and is not affected by the iterative process; the value of ${\mathrm{fitness}}_{\mathrm{food}}$ remains constant. Accordingly, the algorithm only needs to invoke CST once before the entire iterative process is executed in order to obtain the corresponding fitness value, and this value is then placed back into Formula (1) as a constant. In this way, the need to invoke CST at every iteration to obtain the corresponding fitness value is avoided, which substantially saves the simulation time and improves the search efficiency of the algorithm.

The analysis of the simulation time saved by the improved algorithm is as follows. In the original work, suppose that the population size of the algorithm is *N*. In each iteration of the entire algorithm, when the temperature exceeds 30 °C, Formula (11) of the original work, i.e., Formula (1) in this paper, is not invoked; that is, CST is not called for simulation, corresponding to 0 CST calls. Conversely, when the temperature is less than or equal to 30 °C, the invocation of CST is triggered, in which case *N* CST calls are required. Therefore, when computing Formula (1), the number of required CST calls ranges between 0 and *N*. In our improved scheme, however, when computing Formula (1), the part requiring CST invocation is placed outside the loop; consequently, regardless of the temperature, only a single CST invocation is needed. The temperature formula, namely Formula (3) in the original work, is given as Formula (2) below.

(2) temp=rand×15+20

In the above formula, temp denotes the random temperature, and rand denotes a random number in [0, 1]. The original work mentions that the temperature is uniformly distributed between 20 °C and 35 °C. According to the probability formula of the uniform distribution, we can obtain:

(3) $$\begin{array}{cc} P\;\left(\mathrm{temp}>30\right) & =\frac{35-30}{35-20}=\frac{1}{3},\\ P\;\left(\mathrm{temp}\leq 30\right) & =1-P\;\left(\mathrm{temp}>30\right)=\frac{2}{3}. \end{array}\;$$

In Formula (3) above, P(·) denotes the probability. By substituting this probability and the previously analyzed numbers of CST calls into the expectation formula, we obtain the expected number of CST calls per iteration for the original algorithm and the improved algorithm; their computational expressions are given in Formulas (4) and (5) below, respectively.

(4) $$E_{\mathrm{ori}}=0\;\cdot \;\frac{1}{3}+N\;\cdot \;\frac{2}{3}=\frac{2N}{3}$$

(5) $$E_{\mathrm{imp}}=1\;\cdot \;\frac{1}{3}+1\;\cdot \;\frac{2}{3}=1\;$$

In the above expressions, $E_{\mathrm{ori}}$ denotes the expected number of CST software calls per iteration for the original algorithm, and $E_{\mathrm{imp}}$ denotes the expected number of CST software calls per iteration for the improved algorithm. Therefore, the ratio of CST calls that can be saved by the improved algorithm in each iteration is obtained as follows:

(6) $$Rate=\frac{E_{\mathrm{ori}}-E_{\mathrm{imp}}}{E_{\mathrm{ori}}}=1-\frac{1}{E_{\mathrm{ori}}}=1-\frac{3}{2N}\;$$

From Formula (6), it can be seen that as long as the population size of the algorithm exceeds 2, the improved algorithm substantially reduces the number of CST calls; that is, simulation time is saved, thereby improving the execution efficiency of the algorithm. In the two experimental examples presented in this paper, the population sizes are N=20 and N=30, respectively, achieving reductions of 92.5% and 95% in the number of CST calls. In terms of algorithm performance, since the computational parameters involved in evaluating ${\mathrm{fitness}}_{\mathrm{food}}$ are placed outside the loop body, the performance of the algorithm in the original work and that of the improved algorithm are identical.

### 2.3. Transformer Surrogate Model

To achieve the goal of Phase 2 more rapidly and effectively, and to save the algorithm’s exploration time, we chose the approach of co-simulating the algorithm with the surrogate model to search for the optimal solution. After comparing and analyzing various surrogate models, we adopted the Transformer surrogate model because it offers several advantages. First, owing to its self-attention mechanism, it can simultaneously “see” all frequency points and capture long-range resonant couplings, thereby achieving more accurate predictions. Second, it can process the sequence dimension in parallel, so that for a curve consisting of hundreds or thousands of frequency points, the result can be obtained with only a single forward pass, leading to faster processing speed. Third, it can mix input data and output data; for instance, the antenna’s geometric parameters can be treated as additional vector units that interact with the frequency-point vector units, thereby learning the relationship between the antenna geometric structure and the frequency points and better adapting to the high-dimensional geometric design space.

### 2.4. TD3 Reinforcement Learning

The TD3 algorithm, as a widely applied reinforcement learning algorithm, possesses unique advantages. Within a continuous action space, it can perform parameter fine-tuning in local neighborhoods in a small-step, low-oscillation manner. Its dual-Q network can effectively suppress overestimation bias and avoid the occurrence of “false optima,” which is particularly important during local search. Its delayed policy update can reduce search oscillations, making parameter fine-tuning more controllable; meanwhile, its target policy smoothing enables smooth exploration of the neighborhood, making the algorithm less prone to gradient stagnation. In our research, the antenna geometric parameters under investigation are continuous variables, and we aim to achieve the research objective of Phase 2 through local search. Based on the characteristics of the research objective and the advantageous features of the TD3 algorithm, we choose to apply the TD3 algorithm to achieve the research objective.

## 3. Experiments and Analysis

We will use two experimental examples to verify the proposed scheme, with each example employing a different type of microstrip antenna. Experiment 1 will use a slot patch microstrip antenna, and Experiment 2 will use a dual-band, dual-sense circularly polarized slot microstrip antenna. The specifications of the computer and software used throughout the experiments are as follows: 64-bit Windows 11 operating system; Intel(R) Core(TM) Ultra 9 275HX (2.70 GHz) processor; 32.0 GB RAM; NVIDIA GeForce RTX 5070 Ti 16 GB GPU; and CST Studio Suite 2022.

### 3.1. Design and Analysis of Experiment 1

In Experiment 1, we employed a slot patch microstrip antenna to demonstrate the feasibility of the proposed scheme. Based on the initial conditions of the microstrip antenna and the different research stages, we formulated the corresponding research objectives and configured the ICOA, the surrogate model, the TD3 algorithm, and other related components, so that the proposed scheme can effectively achieve the research objectives.

#### 3.1.1. Microstrip Antenna Structure in Experiment 1

**Figure 2:**
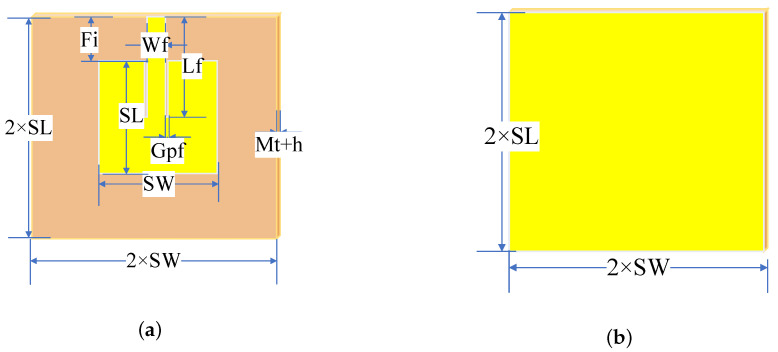
Geometry of the microstrip antenna used in Experiment 1: (**a**) front view and (**b**) back view.

The structure of the microstrip antenna used in Experiment 1 is shown in Figure 2, where the locations of the geometric parameters listed in Table 1 are annotated.

Figure 2a shows the front view of the microstrip antenna structure, and Figure 2b shows the back view of the microstrip antenna structure. The dielectric substrate of the microstrip antenna is made of FR4, with relative permittivity $\varepsilon_{r}=4.3$ and thickness h=4.5 mm; the front patch is made of an ideal conductor with thickness Ct=0.035 mm, and the back patch is made of an ideal conductor with thickness Mt=0.1 mm. The antenna operates over the frequency range of 1.4–2.2 GHz, and its initial geometric dimensions are shown in Table 1.

To ensure that the microstrip antenna achieves impedance matching, i.e., the feed-line impedance of the microstrip antenna is maintained at approximately 50 Ω, and to comply with the microstrip antenna fabrication requirements, the relevant geometric parameters are kept fixed during the research. Specifically, the values of Wf, h, Mt, and Ct are fixed, and the relationship Lf=SL/2−Fi is required to hold. The variation ranges of the remaining parameters are then set according to the objectives of the different phases.

#### 3.1.2. Experiment 1: Phase 1 Experiments and Analysis

In Phase 1, the objective is to use the co-simulation of ICOA with CST to find one or more sets of microstrip antenna geometric parameters such that the operating band ranges from 1.752 GHz to 1.864 GHz, achieving an impedance bandwidth ($S_{11}\leq -10\;\mathrm{dB}$) of 0.112 GHz. The objective function is shown in Formula (7).

(7) $$\begin{array}{cc} \min_{X}\; & \left(S_{11}^{\max}\left(f\right)-\left(-10\right)\right)\\ s.t.\; & 1.752\leq f\leq 1.864\\ \; & lb\leq X\leq ub \end{array}\;$$

In the above formula, $S_{11}^{\max}\left(f\right)$ denotes the maximum $S_{11}$ magnitude within the target frequency band [1.752, 1.864] GHz corresponding to the current antenna geometrical parameters X; −10 denotes the impedance bandwidth threshold, lb and ub denote the lower and upper bounds of the exploration range of the microstrip antenna geometric parameters, respectively.

The first step of the ICOA is to set the exploration range of the population, that is, to set the exploration range of the geometric parameters of the microstrip antenna, as shown in column 2 (ICOA) of Table 2. In column 2 of Table 2, the exploration ranges of SL and SW are ±1.5 times their initial values in Table 1, respectively. The exploration ranges of the other parameters are set based on the structural characteristics of the microstrip antenna and the relationships between the parameters.

Since our proposed ICOA is an improvement based on the COA, and this improvement does not involve the parameters of the original algorithm, we directly adopt the parameters of the original algorithm and only need to set the population size and the number of iterations in the algorithm. Based on the number of microstrip antenna geometric parameters that need to be changed and the number of research objectives, we set the population size to 20 and the number of iterations to 30. (For the specific parameter settings of the algorithm, please refer to [26]).

After setting the above parameters, we performed the co-simulation of ICOA with CST to obtain the corresponding simulation results. When the simulation reached the 13th iteration, the fitness function of the algorithm obtained the optimal value, i.e., 0. The geometric dimensions of the microstrip antenna at this point are shown in Table 3.

**Figure 3:**
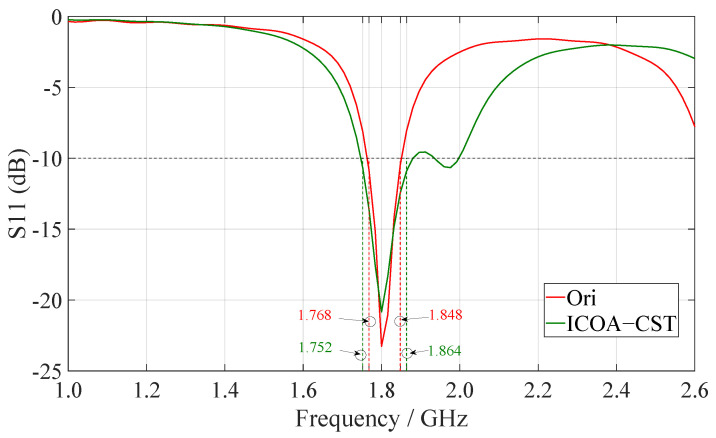
Simulated $S_{11}$ of the microstrip antenna before (Ori) and after Phase 1 optimization (ICOA-CST). Marked frequencies indicate the impedance bandwidth ($S_{11}\leq -10\;\mathrm{dB}$) edges.

Figure 3 shows the microstrip antenna geometric dimensions at the initial state and at Phase 1, along with the $S_{11}\left(\mathrm{dB}\right)$ amplitude curves corresponding to each case. In the figure, the red curve (Ori) is the $S_{11}\left(\mathrm{dB}\right)$ amplitude curve of the microstrip antenna at the initial values, and the green curve (ICOA-CST) is the $S_{11}\left(\mathrm{dB}\right)$ amplitude curve of the microstrip antenna at Phase 1. From the figure, it can be observed that our proposed scheme can achieve the objective of Phase 1.

#### 3.1.3. Experiment 1: Phase 2 Experiments and Analysis

In Phase 2, the objective is to use the co-simulation of TD3 with the Transformer surrogate model to find one or more sets of microstrip antenna geometric parameters that, on the basis of the Phase 1 bandwidth objective, maximize the impedance bandwidth ($S_{11}\leq -10\;\mathrm{dB}$). The objective function is shown in Formula (8).

(8) $$\begin{array}{cc} \max_{X}\; & B_{S_{11}}^{ext}-B_{S_{11}}^{\mathrm{target}\_1}\\ s.t.\; & B_{S_{11}}^{ext}=f_{e2}-f_{e1},\;f_{e2}\geq 1.864,\;f_{e1}\leq 1.752,\\ \; & B_{S_{11}}^{\mathrm{target}\_1}=0.08,\;lb\leq X\leq ub \end{array}\;$$

In the above formula, $B_{S_{11}}^{ext}$ denotes the extended bandwidth (including the Phase 1 target frequency points) under the current microstrip antenna geometric parameters X; $B_{S_{11}}^{\mathrm{target}\_1}$ is is the target bandwidth of Phase 1; and $f_{e2}$ and $f_{e1}$ are are the maximum and minimum frequency points of the Phase 2 target band, respectively.

The sampling range settings in MLHS-Sobol are shown in column 3 (MLHS-Sobol) of Table 2. For the parameters SL, SW, Fi, and Gpf in this column, the upper bounds are approximately 1.25 times the upper bounds in Table 3, and the lower bounds are approximately 0.75 times the lower bounds in Table 3. The main parameter settings are as follows: sample size: 2001; variable dimension: 2; Maximin optimization iterations: 2000. We used this algorithm to collect 2001 sets of microstrip antenna geometric dimension data, which were then fed into CST for simulation to obtain the corresponding $S_{11}\left(\mathrm{dB}\right)$ amplitude values.The above microstrip antenna geometric dimensions and the corresponding $S_{11}\left(\mathrm{dB}\right)$ amplitude values together constitute the dataset for training the Transformer surrogate model, where each data sample consists of 7-dimensional microstrip antenna geometric parameters (SL, SW, h, Ct, Fi, Wf, Gpf) and the $S_{11}\left(\mathrm{dB}\right)$ amplitude values at 101 frequency points. Of the above data, 72% was used as the training set, 8% as the validation set, and 20% as the test set. During the preprocessing of these datasets, the MinMaxScaler method was applied for normalization.

The parameters of the Transformer Encoder surrogate model are set as follows: hidden vector dimension of each encoder layer (d_model): 256; number of heads for multi-head self-attention (nhead): 8; width of the intermediate layer of the feedforward network in the encoder layer (dim_feedforward): 512; dropout ratio of the attention and feedforward in the encoder layer (dropout): 0.1; activation function type of the feedforward network (activation): gelu; number of stacked layers of the encoder layer (num_layers): 4. The input to the surrogate model is the geometric parameters of the microstrip antenna, and the output is a set of predicted $S_{11}\left(\mathrm{dB}\right)$ amplitude values. After the surrogate model is trained, its training loss is 1‰, validation loss is 8%, test loss is 7%, mean squared error (MSE) is 2%, mean absolute error (MAE) is 4.5%, and coefficient of determination ($R^{2}$) is 99.9%. These performance data indicate that the surrogate model can meet the performance requirements.

The exploration range and hyperparameter settings of the TD3 algorithm are shown in column 4 (TD3) of Table 2 and Table 4, respectively. The structural parameters of its Actor network and Critic network are shown in Table 5. In both networks above, the connections between layers are fully connected.

The settings of the state, action, reward, and next state in the environment module of the TD3 algorithm are defined as follows.

(1) **State.** At time *t*, the state $S_{t}$ consists of the current geometrical parameters of the microstrip antenna and the maximum $S_{11}$ value $S_{11,t-1}^{\max}$ over the target frequency band at the previous step, as shown in (9). By incorporating $S_{11,t-1}^{\max}$ into the state, the agent can obtain information about the maximum $S_{11}$ amplitude within the target frequency band.
  (9) $$S_{t}=\left({SL}_{t},{SW}_{t},h_{t},{Ct}_{t},{Fi}_{t},{Wf}_{t},{Gpf}_{t},S_{11,t-1}^{\max}\right)\;$$
(2) **Action.** At time *t*, the action $a_{t}$ is obtained by superimposing the initial action value output by the current policy and the action policy noise, followed by clipping, as shown in (10).
  (10) $$a_{t}=\left(SL_{t}^{\prime },SW_{t}^{\prime },h_{t}^{\prime },Ct_{t}^{\prime },Fi_{t}^{\prime },Wf_{t}^{\prime },Gpf_{t}^{\prime }\right)$$
(3) **Reward.** At time *t*, the reward $r_{t}$, as defined in (11), consists of three components. The first component, $r(\Delta S_{11}^{\max})$, is the reward obtained from the difference between the current maximum $S_{11}$ value $\left(S_{11,t}^{cm}\right)$ within the target frequency band and the previous maximum $S_{11}$ value $\left(S{11,t-1}^{lm}\right)$ of the target frequency band. The second component, $r(S11^{\max})$, is the reward determined by the current maximum $S_{11}$ value within the target frequency band. The third component, r(ΔB), is the reward obtained from the extended bandwidth ΔB on the basis of satisfying the Phase 1 target frequency band. Among these components, the first one constitutes the main part of the reward, the second one represents the absolute performance of the reward, and the third one serves as an additional bonus term.
  (11) $$r_{t}=r\left(\Delta S_{11}^{\max}\right)+r\left(S_{11}^{\max}\right)+r\left(\Delta B\right)\;$$
  The specific mathematical forms of the three reward components in (11) are given in (12).
  (12) $$\begin{array}{cc} r\left(\Delta S_{11}^{\max}\right) & =10\times \left[\left(S_{11,t-1}^{lm}\right)-\left(S_{11,t}^{cm}\right)\right]\\ r\left(S_{11}^{\max}\right) & =0.001\times \left(-S_{11,t}^{cm}\right)\\ r\left(\Delta B\right) & =30\times \left(\Delta B\right) \end{array}$$
(4) **Next State.** The next state is obtained by adding the action to the current state and then clipping the result within the predefined exploration range, as shown in (13). Here, the information component (Info) in the state is updated to $S_{11,t}^{lm}$.
  (13) $$s_{t+1}=s_{t}+a_{t}=\left(SL_{t+1}^{*},SW_{t+1}^{*},h_{t+1}^{*},Ct_{t+1}^{*},Fi_{t+1}^{*},Wf_{t+1}^{*},Gpf_{t+1}^{*},S_{11,t}^{lm}\right)\;$$

Joint simulations using the TD3 algorithm and the Transformer surrogate model yielded numerous sets of microstrip antenna geometrical parameters that satisfied the Phase 2 objective requirements. Among these sets, eight bandwidth-extension cases were achieved, with the maximum bandwidth extension reaching 0.128 GHz. The maximum bandwidth extensions obtained from the simulations are listed in Table 6.

**Figure 4:**
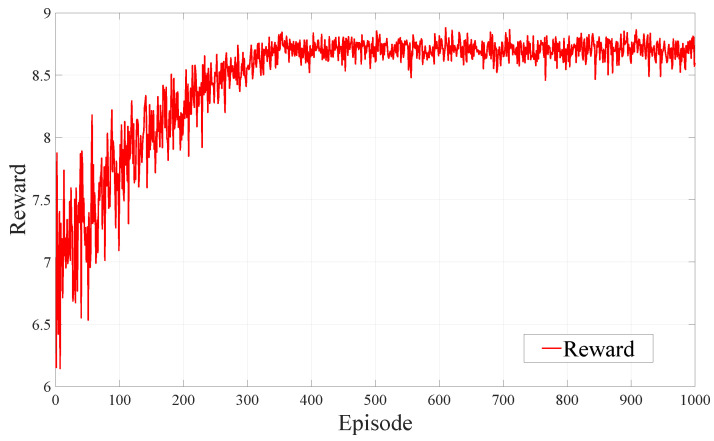
Simulated training reward curve of the TD3 algorithm over 1000 episodes.

Figure 4 shows the reward curve of TD3. As can be observed from the figure, during the early stage of training, i.e., before 350 episodes, the curve changes from large-amplitude oscillations to small-amplitude oscillations. During the later stage of training, i.e., after 350 episodes, the curve tends to become stable. Therefore, it can be considered that the agent has learned an effective policy. In addition, since many sets of microstrip antenna geometrical parameters satisfying the Phase 2 objective were obtained throughout the training process, the training process of the TD3 algorithm can be considered successful.

**Figure 5:**
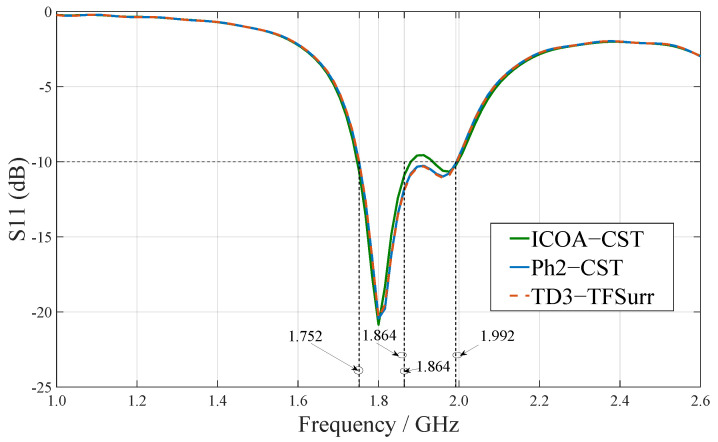
Simulated $S_{11}$ of the microstrip antenna across the two-phase optimization: ICOA-CST (Phase 1, CST simulation), TD3-TFSurr (Phase 2, surrogate-model prediction), and Ph2-CST (CST re-simulation of the Phase 2 geometry for verification). Marked frequencies indicate the impedance bandwidth ($S_{11}\leq -10\;\mathrm{dB}$) edges.

Figure 5 shows the $S_{11}\left(\mathrm{dB}\right)$ curve obtained in Stage 1, as represented by the solid green line (ICOA-CST); the $S_{11}\left(\mathrm{dB}\right)$ curve corresponding to the maximum bandwidth extension in Stage 2, as represented by the dashed orange line (TD3-TFSurr); and the $S_{11}\left(\mathrm{dB}\right)$ curve obtained by simulating the microstrip antenna geometrical parameters obtained in Stage 2 using CST software, as represented by the solid blue line (Ph2-CST). By comparing the bandwidth of the dashed orange line with that of the solid green line, it can be observed that the algorithm achieves the Stage 2 objective. In addition, the similar variation trends of the dashed orange line and the solid blue line indicate that the Transformer Encoder surrogate model can accurately predict the $S_{11}\left(\mathrm{dB}\right)$ values.

Table 7 summarizes the bandwidth extension results in the two stages. As shown in the table, after the two-stage extension process, the obtained bandwidth is 0.16 GHz larger than the original bandwidth, corresponding to an extension ratio of 200%.

**Figure 6:**
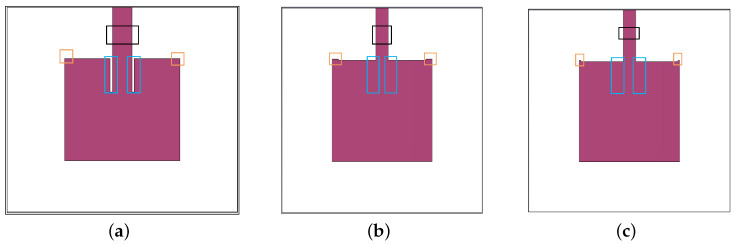
Evolution of the microstrip antenna layout: (**a**) original design, (**b**) geometry after Phase 1 optimization, and (**c**) geometry after Phase 2 optimization. Colored rectangles (black, orange, blue) highlight the regions whose dimensions are modified between consecutive stages.

Figure 6 presents the front views of the microstrip antenna in three cases: the original design, as shown in Figure 6a; the design after Phase 1 optimization, as shown in Figure 6b; and the design after Phase 2 optimization, as shown in Figure 6c. The main differences among the three layouts are highlighted using rectangular boxes with different colors. The geometry on the back side of the microstrip antenna remains unchanged.

We compared the proposed algorithm (ICOA-TD3) with four other algorithms, namely GA, PSO, DE, and Bayesian Optimization (BO). To ensure the fairness of the comparison, the same population size, number of iterations, fitness function, and surrogate model were used for these three algorithms. First, joint simulations were performed using each algorithm and the surrogate model. Then, the microstrip antenna geometrical parameters corresponding to the fitness values obtained from the simulations were verified using CST software to ensure the reliability of the fitness values.

**Figure 7:**
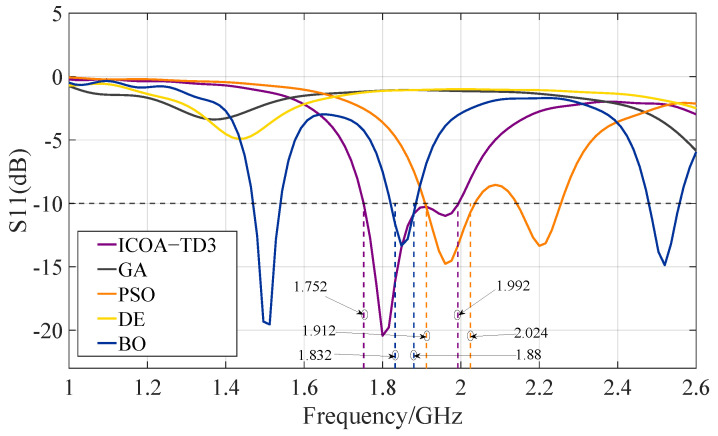
Simulated $S_{11}$ of the microstrip antenna optimized by four algorithms: the proposed ICOA-TD3, GA, PSO, DE, and BO. Marked frequencies indicate the impedance bandwidth ($S_{11}\leq -10\;\mathrm{dB}$) edges.

Figure 7 shows the $S_{11}\left(\mathrm{dB}\right)$ curves of different algorithms after the optimization process is completed. The ICOA-TD3 curve in this figure is obtained from the Ph2-CST result shown in Figure 5. Table 8 summarizes the bandwidth increases achieved by these algorithms, with the optimal value highlighted in bold. As can be observed from Figure 7, compared with the other three algorithms, the proposed algorithm (ICOA-TD3) can achieve $S_{11}$ bandwidth extension on the basis of the original frequency band. Table 8 further shows that the proposed algorithm achieves the largest bandwidth increase.

### 3.2. Design and Analysis of Experiment 2

The results obtained from Experiment 1 verify the feasibility of the proposed scheme. To further validate its feasibility, Experiment 2 is conducted. In Experiment 2, the dual-band dual-sense circularly polarized slot microstrip antenna reported in [28] is employed. This antenna involves two types of bandwidth in two frequency bands, namely the impedance bandwidth ($S_{11}\leq -10\;\mathrm{dB}$) and AR bandwidth in the low-frequency band, and the impedance bandwidth ($S_{11}\leq -10\;\mathrm{dB}$) and AR bandwidth in the high-frequency band.

#### 3.2.1. Microstrip Antenna Structure in Experiment 2

**Figure 8:**
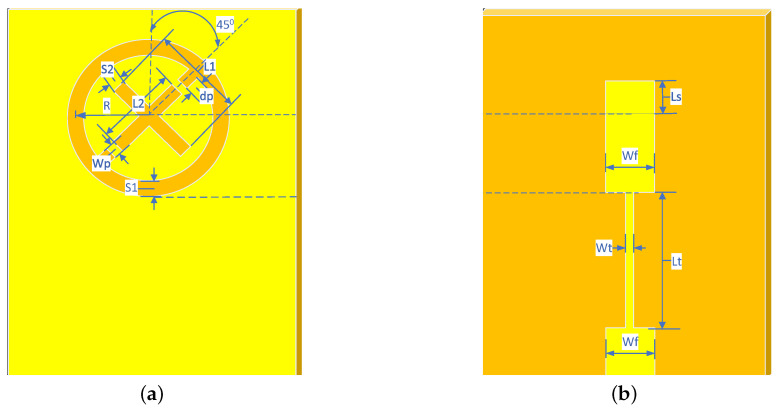
Geometry of the microstrip antenna used in Experiment 2: (**a**) front view and (**b**) back view.

The structure of the microstrip antenna used in Experiment 2 is shown in Figure 8, in which the locations of the geometrical parameters listed in Table 9 are indicated.

Figure 8a shows the front view of the microstrip antenna structure, and Figure 8b shows the back view. The substrate of the microstrip antenna has a relative permittivity of $\epsilon_{r}=4.2$ and a thickness of h=1.45mm. The front and back patches are modeled as perfect electric conductors with a metal thickness of 0.035mm. The initial geometrical dimensions of the antenna are listed in Table 9. The lengths L1 and L2 satisfy the relationship dl=L1−L2. To ensure an impedance matching of approximately 50 Ω, the values of the parameters S1, Wt, and Wf are kept constant.

#### 3.2.2. Experiment 2: Phase 1 Experiments and Analysis

In Phase 1, the objective is to employ the ICOA in conjunction with CST co-simulation to identify one or more sets of microstrip antenna geometries that satisfy the specified impedance bandwidth ($S_{11}\leq -10\;\mathrm{dB}$) target ($B_{S_{11}}^{\mathrm{target}}$) and AR bandwidth target ($B_{\mathrm{AR}}^{\mathrm{target}}$). Specifically, the impedance bandwidth ($S_{11}\leq -10\;\mathrm{dB}$) target $B_{s,l}$ in the low-frequency band $f_{s,l}$ is defined over the frequency range from 2.12 GHz to 2.64 GHz, while the impedance bandwidth ($S_{11}\leq -10\;\mathrm{dB}$) target $B_{s,h}$ in the high-frequency band $f_{s,h}$ is defined over the frequency range from 5.24 GHz to 6.70 GHz. For the AR bandwidth, the low-frequency bandwidth target $B_{a,l}$ in $f_{a,l}$ is to achieve a bandwidth of 0.16 GHz within the 2–3 GHz band, and the high-frequency bandwidth target $B_{a,h}$ in $f_{a,h}$ is to achieve a bandwidth of 0.06 GHz within the 6–7 GHz band. Equation (14) defines the Phase 1 objective function, which also serves as the fitness function of the algorithm.


(14) $$\begin{array}{cc} \min_{X}\; & \left(\frac{1}{20}\sum_{i=1}^{2}\max_{i}\;\left(0,\;S_{11}^{\max}\left(f\right)-\left(-10\right)\right)+\frac{1}{0.22}\sum_{i=1}^{2}\max_{i}\;\left(0,\;B_{\mathrm{AR}}^{\mathrm{target}}-B_{\mathrm{AR}}^{\max}\left(f;X\right)\right)\right)\\ s.t.\; & 2.12\leq f_{s,l}\leq 2.64,\;5.24\leq f_{s,h}\leq 6.70,\\ \; & 2.00\leq f_{a,l}\leq 3.00,\;6.00\leq f_{a,h}\leq 7.00,\\ \; & lb\leq X\leq ub. \end{array}\;$$

In the above formula, $S_{11}^{\max}\left(f\right)$ represents the maximum $S_{11}\left(\mathrm{dB}\right)$ value within the $f_{s,l}$ or $f_{s,h}$ frequency band for the current antenna geometry X; −10 is the threshold for determining the impedance bandwidth. $B_{\mathrm{AR}}^{\mathrm{target}}$ denotes the prescribed AR target bandwidth within the $f_{a,l}$ or $f_{a,h}$ frequency band, and $B_{\mathrm{AR}}^{\max}$ denotes the maximum AR bandwidth within $f_{a,l}$ or $f_{a,h}$ for the current antenna geometry X. $\max_{i},\left(\;\cdot \;\right)$ represents the maximum value among the candidate values in (·), and *i* denotes the low- and high-frequency-band cases. The coefficients $\frac{1}{20}$ and $\frac{1}{0.22}$ are used to normalize the summed terms in the formula, thereby ensuring that $B_{S_{11}}^{\mathrm{target}}$ and $B_{\mathrm{AR}}^{\mathrm{target}}$ have equal search weights in the algorithm. lb and ub are the lower and upper limits of the antenna geometry exploration range, respectively.

Based on the characteristics and research objectives of the microstrip antenna, the exploration range for the antenna geometrical dimensions is set as shown in column 2 (ICOA) of Table 10. To ensure that the antenna geometrical dimensions are reasonable, the following constraint is imposed: R−0.5−dp−0.5×(L1−dl)≥0.1.

The parameters of the ICOA used in this experiment are kept the same as those in the original algorithm. The population size is set to 30, and the number of iterations is set to 30. After all iterations were completed, a relatively high fitness value, i.e., a relatively good solution to the objective function, was obtained, with a value of **0.210756**. The corresponding microstrip antenna geometry is shown in Table 11.

**Figure 9:**
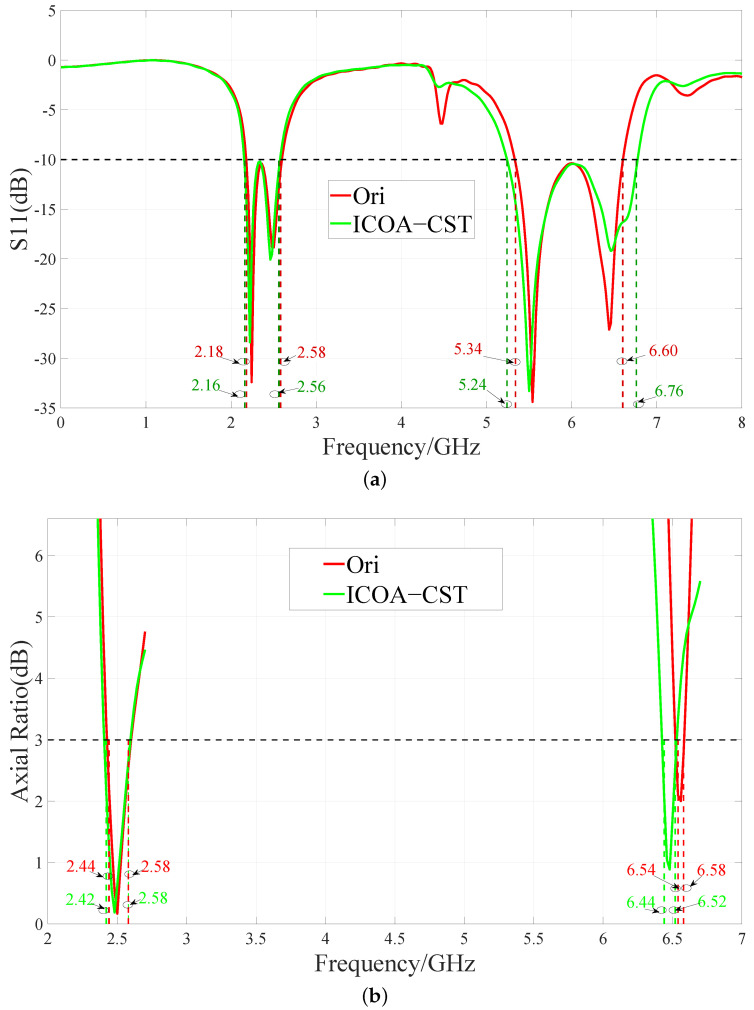
Simulated (**a**) $S_{11}$ and (**b**) AR of the dual-band microstrip antenna before (Ori) and after Phase 1 optimization (ICOA-CST). Marked frequencies indicate the impedance bandwidth ($S_{11}\leq -10\;\mathrm{dB}$) edges in (**a**) and the AR bandwidth (AR≤3dB) edges in (**b**).

Figure 9 shows the $S_{11}\left(\mathrm{dB}\right)$ amplitude curves (Figure 9a) and AR (dB) amplitude curves (Figure 9b) corresponding to the microstrip antenna geometries in the initial state and after Phase 1 optimization, respectively. In Figure 9a, the red curve represents the $S_{11}\left(\mathrm{dB}\right)$ curve of the microstrip antenna with the initial geometry, while the green curve represents the $S_{11}\left(\mathrm{dB}\right)$ curve with the optimized geometry. It can be seen from the figure that the bandwidth extension target is achieved in the high-frequency band, while the bandwidth in the low-frequency band remains within the original bandwidth range. A similar conclusion can be drawn from Figure 9b; that is, in the high-frequency band, the AR bandwidth of the green curve is wider than that of the red curve, whereas the bandwidths in the low-frequency band are nearly the same. Therefore, it can be concluded that in the high-frequency band the algorithm can achieve the specified bandwidth target, while in the low-frequency band it can maintain the bandwidth within the original bandwidth range.

#### 3.2.3. Experiment 2: Phase 2 Experiments and Analysis

In Phase 2, the objective is to employ the TD3 algorithm in conjunction with the Transformer surrogate model in a co-simulation framework to search for one or more sets of microstrip antenna geometries. On the basis of the Phase 1 bandwidth targets, the impedance bandwidth ($S_{11}\leq -10\;\mathrm{dB}$) and AR bandwidth are further maximized. Equation (15) defines the Phase 2 objective function.

(15) $$\begin{array}{cc} \max_{X}\; & \left(\frac{1}{1.92}\sum_{i=1}^{2}\max\;\left(0,\;B_{S_{11}}^{\mathrm{ext}}-B_{S_{11}}^{\mathrm{target}\_1}\right)+\frac{1}{0.22}\sum_{i=1}^{2}\max\;\left(0,\;B_{\mathrm{AR}}^{\mathrm{ext}}-B_{\mathrm{AR}}^{\mathrm{target}\_1}\right)\right)\\ s.t.\; & f_{e1}\leq 2.16\leq f_{s,l}\leq 2.56\leq f_{e2},\;f_{e3}\leq 5.24\leq f_{s,h}\leq 6.76\leq f_{e4}\\ \; & 2\leq f_{a,l}\leq 3,\;6\leq f_{a,h}\leq 7\\ \; & lb\leq X\leq ub \end{array}\;$$

In the above formula, $B_{S11}^{\mathrm{ext}}$ represents the extended impedance bandwidth ($S_{11}\leq -10\;\mathrm{dB}$) within the low- or high-frequency band $f_{s,l}$ or $f_{s,h}$ for the current antenna geometry X, and $B_{S11}^{\mathrm{target}\_1}$ denotes the Phase 1 S11 target bandwidth in $f_{s,l}$ or $f_{s,h}$. The symbols $f_{e2}$ and $f_{e1}$ denote the upper and lower frequency limits, respectively, of the Phase 2 $S_{11}$ target in the low-frequency band, while $f_{e4}$ and $f_{e3}$ denote the upper and lower frequency limits, respectively, in the high-frequency band. $B_{\mathrm{AR}}^{\mathrm{ext}}$ represents the extended AR bandwidth within the low- or high-frequency band $f_{a,l}$ or $f_{a,h}$ for the current antenna geometry X, and $B_{\mathrm{AR}}^{\mathrm{target}\_1}$ denotes the Phase 1 AR target bandwidth in $f_{a,l}$ or $f_{a,h}$. Similarly, the coefficients $\frac{1}{1.92}$ and $\frac{1}{0.22}$ are introduced to normalize the $S_{11}$ and AR extended-bandwidth terms, ensuring that both terms have equal search weights in the algorithm.

Column 3 of Table 10 (MLHS-Sobol) indicates the range of microstrip antenna geometrical dimensions used for MLHS-Sobol sampling. In this experiment, 3000 sets of microstrip antenna geometrical-dimension data were collected and fed into CST for simulation to obtain the corresponding $S_{11}\left(\mathrm{dB}\right)$ and AR (dB) amplitude values. These microstrip antenna geometrical-dimension data and their corresponding amplitude values together constitute the dataset for training the Transformer Encoder surrogate model. This dataset was divided into three subsets: 70% for training, 15% for validation, and 15% for testing. During data preprocessing, the MinMaxScaler method was applied for normalization.

Column 4 (TD3) of Table 10 lists the exploration range of the microstrip antenna geometrical parameters in the TD3 algorithm. The main hyperparameters of TD3 in this experiment are generally consistent with those used in Experiment 1, i.e., the parameters listed in Table 4, except that the target policy noise and noise clip are set to 0.2 and 0.3, respectively. The structural parameters of the Actor network and Critic network in TD3 are also generally consistent with those in Experiment 1. The only differences are that the input to the first layer of the Actor network is 12-dimensional state information, and the output of its third layer is 8-dimensional action information; for the Critic network, the input to the first layer is 20-dimensional information formed by combining the state and action information.

In this experiment, since two types of bandwidth extension are involved, the state, action, reward, and next state components of the environment module in the algorithm are relatively complex. After comparing the results of multiple experiments, these components were defined as follows:

(1) **State.** The current state $S_{t}$, as defined in (16), consists of the current antenna geometrical parameters; the maximum $S_{11}$ values within the target low- and high-frequency bands at the previous time step ($S_{11,t-1}^{\max,\mathrm{lf}}$, $S_{11,t-1}^{\max,\mathrm{hf}}$); and the AR bandwidths in the low- and high-frequency bands at the previous time step ($B_{\mathrm{AR},t-1}^{\mathrm{lf}}$, $B_{\mathrm{AR},t-1}^{\mathrm{hf}}$).
  (16) $$S_{t}=\left(R_{t},{L1}_{t},{S2}_{t},{dp}_{t},{Wp}_{t},{Ls}_{t},{Lt}_{t},{dl}_{t},S_{11,t-1}^{\max,\mathrm{lf}},S_{11,t-1}^{\max,\mathrm{hf}},B_{\mathrm{AR},t-1}^{\mathrm{lf}},B_{\mathrm{AR},t-1}^{\mathrm{hf}}\right)$$
(2) **Action.** The action $a_{t}$ here is shown in Formula (17), which is similar to the action in Experiment 1, except that the value in column 4 (TD3) of Table 10 is used in the clipping range.
  (17) $$a_{t}=\left(R_{t}^{\prime },L1_{t}^{\prime },S2_{t}^{\prime },dp_{t}^{\prime },Wp_{t}^{\prime },Ls_{t}^{\prime },Lt_{t}^{\prime },dl_{t}^{\prime }\right)$$
(3) **Reward.** The algorithm’s reward $r_{t}$, as defined in (18), consists of the $S_{11}$ reward term $r(S_{11})$ and the AR bandwidth reward term $r(\Delta B_{\mathrm{AR}})$. The overall reward is mainly constructed from bandwidth differences. It is evaluated over two frequency bands (low and high), and the same reward formulation is applied to both bands, i.e., they share an identical reward function.
  (18) $$r_{t}=r\left(S_{11}\right)+r\left(\Delta B_{\mathrm{AR}}\right)$$
  The specific composition of the reward $r(S_{11})$ in the above formula is shown in (19). It consists of the impedance bandwidth ($S_{11}\leq -10\;\mathrm{dB}$)-increment reward $r(\Delta B_{S_{11}})$ and the term $r(-\alpha \;S_{11}^{\max})$, which is proportional to the maximum $S_{11}$ value within the frequency band when no bandwidth extension is achieved.
  (19) $$r\left(S_{11}\right)=r\left(\Delta B_{S_{11}}\right)+r\left(-\alpha S_{11}^{\max}\right)\;$$
  The specific composition of reward $r(\Delta B_{S_{11}})$ in the above formula is shown in (20), and it is defined as a three-case piecewise reward. The first case assigns a reward of “+1” when the current bandwidth $B_{S_{11}}^{t}$ is greater than or equal to the Phase 1 target bandwidth $B_{S_{11}}^{\mathrm{target}}$. The second case applies a penalty of “−0.5” when $B_{S_{11}}^{t}$ is less than $B_{S_{11}}^{\mathrm{target}}$ but greater than or equal to the previous bandwidth $B_{S_{11}}^{t-1}$. The third case applies a penalty of “−1” in all other situations.
  (20) $$r\left(\Delta B_{S_{11}}\right)=\left\{\begin{array}{cc} 1 & B_{S_{11}}^{t}\geq B_{S_{11}}^{\mathrm{target}}\\ -0.5 & \left(B_{S_{11}}^{t}\geq B_{S_{11}}^{t-1}\right)\wedge \left(B_{S_{11}}^{t}<B_{S_{11}}^{\mathrm{target}}\right)\\ -1 & \mathrm{otherwise} \end{array}\right.\;$$
  The specific composition of reward $r(-\alpha \;S_{11}^{\max})$ in Formula (19) is shown in (21).This term is introduced to provide an additional positive incentive, helping the agent better learn the desired policy. A small positive reward is assigned when $B_{S_{11}}^{t}$ is smaller than $B_{S_{11}}^{\mathrm{target}}$.
  (21) $$r\left(-\alpha S_{11}^{\max}\right)=-0.08\times S_{11,t}^{\max}$$
  The specific composition of the AR bandwidth reward term $r(\Delta B_{\mathrm{AR}})$ in Formula (18) is given in Formula (22), and it also adopts a three-case piecewise definition. The first case assigns a reward of “+1” when the current bandwidth $B_{\mathrm{AR}}^{t}$ is greater than or equal to the Phase 1 target bandwidth $B_{\mathrm{AR}}^{\mathrm{target}}$. The second case assigns a reward of “0” (i.e., no additional reward or penalty) when $B_{\mathrm{AR}}^{t}$ is less than $B_{\mathrm{AR}}^{\mathrm{target}}$ but greater than or equal to the previous bandwidth $B_{\mathrm{AR}}^{t-1}$. The third case applies a penalty of “−1” in all other situations.
  (22) $$r\left(\Delta B_{\mathrm{AR}}\right)=\left\{\begin{array}{cc} 1 & B_{\mathrm{AR}}^{t}\geq B_{\mathrm{AR}}^{\mathrm{target}}\\ 0 & \left(B_{\mathrm{AR}}^{t}\geq B_{\mathrm{AR}}^{t-1}\right)\wedge \left(B_{\mathrm{AR}}^{t}<B_{\mathrm{AR}}^{\mathrm{target}}\right)\\ -1 & \mathrm{otherwise} \end{array}\right.\;$$
(4) **Next state.** The next state $s_{t+1}$, as defined in (23), consists of three components: (i) the new antenna geometry obtained by adding the action to the current state and clipping the result to the predefined exploration range; (ii) the maximum $S_{11}$ values within the target low- and high-frequency bands at the current time step ($S_{11,t}^{\max,\mathrm{lf}}$, $S_{11,t}^{\max,\mathrm{hf}}$); and (iii) the AR bandwidths in the low- and high-frequency bands at the current time step ($B_{\mathrm{AR},t}^{\mathrm{lf}}$, $B_{\mathrm{AR},t}^{\mathrm{hf}}$).
  (23) $$S_{t+1}=\left(R_{t+1}^{*},L1_{t+1}^{*},S2_{t+1}^{*},dp_{t+1}^{*},Wp_{t+1}^{*},Ls_{t+1}^{*},Lt_{t+1}^{*},dl_{t+1}^{*},S_{11,t}^{\max,\mathrm{lf}},S_{11,t}^{\max,\mathrm{hf}},B_{\mathrm{AR},t}^{lf},B_{\mathrm{AR},t}^{hf}\right)$$

During the co-simulation of TD3 and the Transformer surrogate model, multiple relatively good sets of microstrip antenna geometries were identified. These geometries can achieve simultaneous bandwidth extension in up to three bandwidth categories, namely the high-frequency band ($S_{11}$_high) of $S_{11}$, the low-frequency band of AR (AR_low), and the high-frequency band of AR (AR_high). However, bandwidth extension in the low-frequency band ($S_{11}$_low) of $S_{11}$ was not achieved. The maximum bandwidth extension values are 0.08 GHz for $S_{11}$_high, 0.02 GHz for AR_low, and 0.06 GHz for AR_high. Table 12 lists the microstrip antenna geometries obtained from the simulation when the maximum bandwidth extension is achieved.

**Figure 10:**
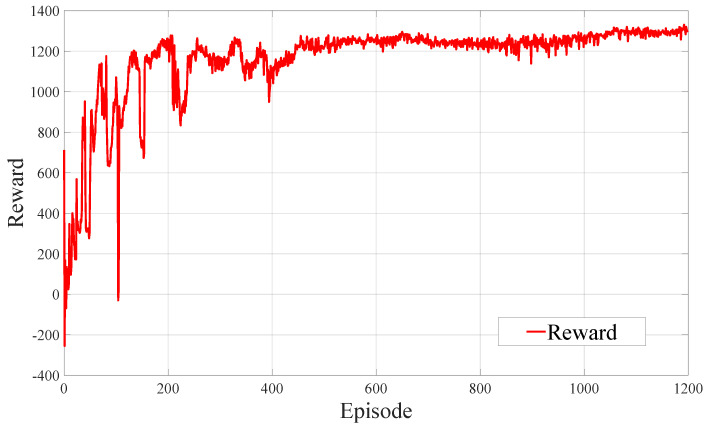
Simulated training reward curve of the TD3 algorithm over 1200 episodes.

Figure 10 shows the reward curve obtained from the joint simulation of TD3 and the Transformer surrogate model in this experiment. As can be seen from the figure, in the early stage of training, i.e., before 450 episodes, the curve changes from large-amplitude oscillations to small-amplitude oscillations. In the later stage of training, i.e., after 450 episodes, the curve becomes relatively stable. Therefore, it can be concluded that the agent has effectively learned the policy. Combined with the fact that many sets of microstrip antenna geometries satisfying the Phase 2 objective were obtained during the simulation process, it can be concluded that the overall training process was successful.

**Figure 11:**
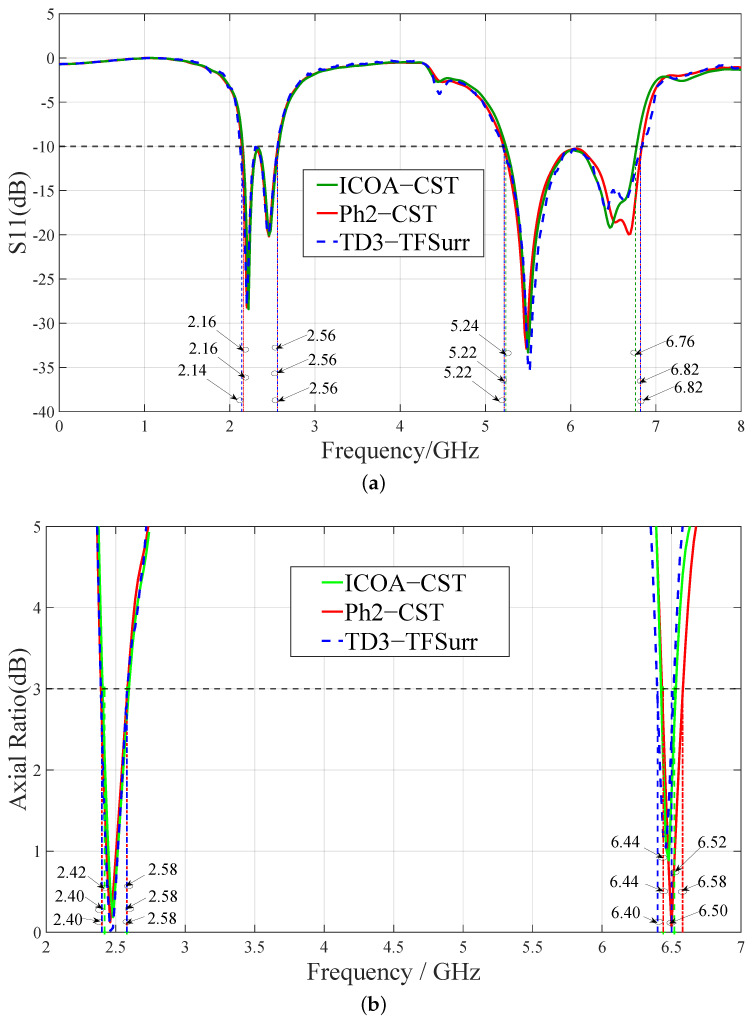
Simulated (**a**) $S_{11}$ and (**b**) AR of the dual-band microstrip antenna across the two-phase optimization: ICOA-CST (Phase 1, CST simulation), TD3-TFSurr (Phase 2, surrogate-model prediction), and Ph2-CST (CST re-simulation of the Phase 2 geometry for verification). Marked frequencies indicate the impedance bandwidth ($S_{11}\leq -10\;\mathrm{dB}$) edges in (**a**) and the AR bandwidth (AR≤3dB) edges in (**b**).

In Figure 11a and Figure 11b, the solid green line (ICOA-CST) represents the $S_{11}\left(\mathrm{dB}\right)$ and AR (dB) curves obtained from the co-simulation of the ICOA and CST in Phase 1, respectively; the dashed blue line (TD3-TFSurr) represents the curves predicted by the Transformer surrogate model for a set of microstrip antenna geometries identified by the TD3 algorithm in Phase 2; and the solid red line (Ph2-CST) represents the $S_{11}\left(\mathrm{dB}\right)$ and AR (dB) curves obtained by substituting this set of geometrical dimensions into CST for simulation. Furthermore, the dashed blue lines in Figure 11a,b exhibit wider bandwidths than the solid green lines (ICOA-CST). In addition, the frequency points of the dashed blue line in Figure 11a include the frequency points corresponding to the Phase 1 bandwidth represented by the solid green line. Therefore, the algorithm achieved most of the Phase 2 objectives, except for the objective in the low-frequency band of $S_{11}$. Similarly, the solid red line and dashed blue line in Figure 11a show similar trends, and the two curves in the low-frequency band of Figure 11b also show similar trends. However, in the high-frequency band of Figure 11b, there is a deviation between the two curves in terms of the frequency-point positions. Therefore, it can be concluded that although there are discrepancies between the predicted values of the surrogate model and the actual values, the surrogate model still exhibits good predictive capability.

Table 13 summarizes the bandwidth extension achieved in the two phases, based on the bandwidth extension data extracted from Figure 9 and Figure 11. The table shows that, after the two-phase bandwidth extension process, the obtained bandwidths increased by 0 GHz, 0.34 GHz, 0.04 GHz, and 0.1 GHz compared with the original bandwidths, corresponding to extension ratios of 0%, 27%, 28%, and 250%, respectively.

**Figure 12:**
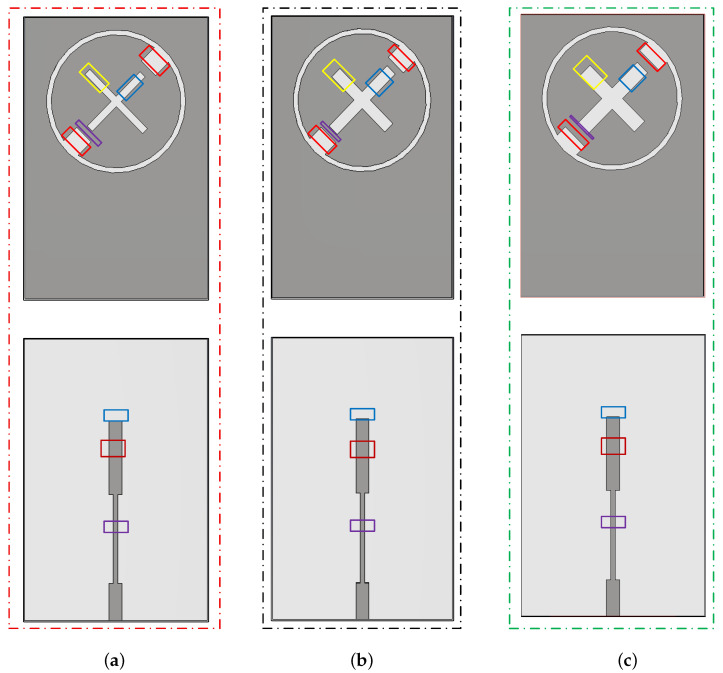
Evolution of the microstrip antenna layout: (**a**) original design, (**b**) geometry after Phase 1 optimization, and (**c**) geometry after Phase 2 optimization. Colored rectangles (red, yellow, blue, purple) highlight the regions whose dimensions are modified between consecutive stages.

Figure 12 shows the structural changes on the front and back sides of the antenna. Figure 12a depicts the original antenna structure; Figure 12b shows the structure after Phase 1 optimization; and Figure 12c shows the structure after Phase 2 optimization. Throughout the figure, rectangular markers in different colors are used to highlight the main differences among the three structural layouts.

As in Experiment 1, the proposed algorithm (ICOA-TD3) was compared with three other algorithms, namely GA, PSO, DE, and BO. The experimental settings for the comparison were kept consistent with those used in Experiment 1.

**Figure 13:**
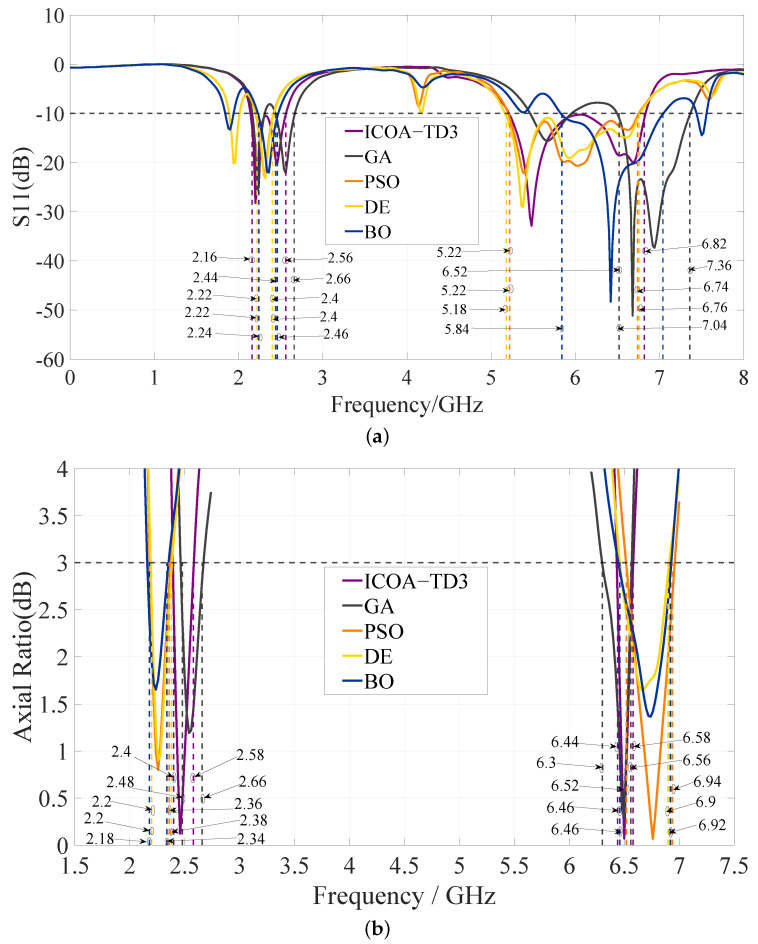
Simulated (**a**) $S_{11}$ and (**b**) AR of the dual-band microstrip antenna optimized by four algorithms: the proposed ICOA-TD3, GA, PSO, DE and BO. Marked frequencies indicate the impedance bandwidth ($S_{11}\leq -10\;\mathrm{dB}$) edges in (**a**) and the AR bandwidth (AR≤3dB) edges in (**b**).

Figure 13 shows the $S_{11}\left(\mathrm{dB}\right)$ and AR(dB) curves of different algorithms after the completion of the optimization process. The ICOA-TD3 curve in the figure is obtained from the Ph2-CST result shown in Figure 11. Table 14 shows the bandwidth increase of these algorithms, with the optimal values highlighted in bold. Figure 13 shows that only our proposed algorithm (ICOA-TD3) can guarantee bandwidth expansion of $S_{11}$ based on the original frequency. This expansion includes both $S_{11}$ and AR bandwidth. Furthermore, Table 14 shows that our proposed algorithm achieves the most bandwidth increments.

## 4. Results and Discussion

Table 7 and Figure 5 present the results obtained in Phase 1 and Phase 2 of Experiment 1, respectively. Similarly, Table 13 and Figure 11 present the results obtained in Phase 1 and Phase 2 of Experiment 2, respectively. These tables and figures demonstrate the feasibility of the proposed method. In Experiment 1, the geometrical dimensions of the microstrip antenna fully satisfied the objective requirements of both phases, indicating that the optimal solution of the fitness function was obtained. In Experiment 2, three out of the four types of bandwidth extension were realized. Although the low-frequency-band impedance bandwidth ($S_{11}\leq -10\;\mathrm{dB}$) was not extended, it did not exhibit significant narrowing and remained essentially unchanged. There are two main reasons for this unmet objective. First, the bandwidth of a microstrip antenna is constrained by its inherent physical characteristics, which impose an upper limit on the achievable bandwidth. Second, the extended bandwidth terms are interrelated and mutually affected; therefore, certain trade-offs and constraints exist among them during the bandwidth extension process.

The proposed method for extending the bandwidth of microstrip antennas has two main advantages. First, it enables bandwidth extension within the original frequency bands of the microstrip antenna, and the two-phase extension process provides sufficient bandwidth margin. Second, the proposed ICOA can improve the search efficiency, while the Transformer Encoder surrogate model used in the algorithm achieves high prediction accuracy. The main limitations of this method are as follows. First, each co-simulation involving the ICOA and CST software is time-consuming. Second, establishing the surrogate model also requires considerable time. In future research, methods for reducing the simulation time will be further investigated to achieve more efficient bandwidth extension of microstrip antennas.

## 5. Conclusions

This paper investigates the bandwidth extension of microstrip antennas and proposes a method that combines an improved crayfish optimization algorithm, a Transformer surrogate model, and TD3 reinforcement learning. The feasibility of the proposed method is validated through two experiments. In addition, the proposed algorithm can also be applied to optimization problems in other engineering applications. Although the proposed algorithm demonstrates good performance in enhancing antenna bandwidth, experimental validation using fabricated antenna prototypes and measurements has not yet been conducted. Practical factors, such as fabrication tolerances, material uncertainties, and measurement environments, may affect antenna bandwidth performance. Future work will focus on the fabrication of an antenna prototype, experimental validation, and robustness analysis to further evaluate the practical applicability of the proposed method.

## Figures and Tables

**Table 1:** Initial geometrical dimensions of the microstrip antenna in Experiment 1.

Parameter	SL	SW	Lf	Wf	h	Fi	Gpf	Mt	Ct
Value ^1^	38	51	31.5	8.7	4.5	12.5	1	0.1	0.035

^1^ All dimensions are in mm.

**Table 2:** Exploration ranges of microstrip antenna geometrical parameters for each stage in Experiment 1.

Parameter	Value of Exploration Range ^1^		
	ICOA	MLHS–Sobol	TD3
SL	(19,57)	(28.05,46.74)	(29.9,44.87)
SW	(25.5,76.5)	(52.26,87.1)	(55.75,83.62)
Fi	(0.5,SL−0.1)	(0.375,0.625)	(0.4,0.6)
Gpf	$\left(0.1,\;(\mathrm{SW}-\mathrm{Wf})/2\right)$	(19.52,32.53)	(20.82,31.23)

^1^ All dimensions are in mm.

**Table 3:** Microstrip antenna geometrical parameters obtained in Phase 1 of Experiment 1.

Parameter	Value ^1^
SL	37.3935713
SW	69.6828155
Fi	0.5
Gpf	26.0261541

^1^ All dimensions are in mm.

**Table 4:** Main hyperparameter settings for the TD3 algorithm.

Parameter	Value
learning rate of Actor or Critic	$3\times 10^{-4}$
discount factor γ	0.99
soft update parameter τ	0.005
policy delay update frequency	2
target policy noise	0.3
noise clip	0.5
batch size	256
memory size	$1\times 10^{6}$
number of episodes	1000
number of steps in each episode	500

**Table 5:** Network structure parameters of Actor network and Critic network in TD3.

Network Type	Connection Type	Input Layer Count	Output Layer Count	Activation Type
Actor network	layer 1: fully connected layer	8	256	ReLU
	layer 2: fully connected layer	256	256	ReLU
	layer 3: fully connected layer	256	7	Tanh
Critic network 1 or Critic network 2	layer 1: fully connected layer	15	256	ReLU
	layer 2: fully connected layer	256	256	ReLU
	layer 3: fully connected layer	256	1	/

**Table 6:** Microstrip antenna geometry obtained in Phase 2 of Experiment 1.

Parameter	SL	SW	Fi	Gpf
Value ^1^	37.31924104	69.80663924	0.44353042	29.30228411

^1^ All dimensions are in mm.

**Table 7:** Statistics of bandwidth expansion of Experiment 1.

Parameter	Original Bandwidth (GHz)	Bandwidth Extension of Phase 1 (GHz)	Bandwidth Extension of Phase 2 (GHz)	Total Bandwidth Extension (GHz)	Total Bandwidth Extension (%)
Value	0.08	0.032	0.128	0.16	200%

**Table 8:** Statistics on the increase in bandwidth for different algorithms in Experiment 1.

Parameter	ICOA-TD3 ^1^	GA	PSO	DE	BO
Value ^2^	**0.16**	−0.08	0.032	−0.08	−0.032

^1^ Its value is the data in column 5 (Total Bandwidth Extension) of Table 7. ^2^ All dimensions are in GHz.

**Table 9:** Initial geometrical dimensions of the microstrip antenna in Experiment 2.

Parameter	R	L1	S2	dp	Wp	S1	Ls	Wf	Lt	Wt	dl
Value ^1^	14	17	1.5	4	4.2	1	0.4	2.8	18	0.96	1.5

^1^ All dimensions are in mm.

**Table 10:** Exploration ranges of microstrip antenna geometrical parameters for each stage in experiment 2.

Parameter	Value of Exploration Range ^1^		
	ICOA	MLHS–Sobol	TD3
R	(13,17)	(8.1633,20.1633)	(9.91431,18.41229)
L1	(15,19)	(10.0,22.0)	(11.2,20.8)
S2	(1,3.5)	(0.1,8.4353)	(1.70471,3.16589)
dp	(2.5,5.5)	(0.1,9.5)	(2.45,4.55)
Wp	(6,7.5)	(0.1,10.5)	(3.15,5.85)
Ls	(0.3,2)	(−5.446,6.554)	(0.3878,0.7202)
Lt	(16,20)	(12.3385,24.3385)	(12.83695,23.84005)
dl	(−1.5,3)	(−7.5,4.5)	(−1.95,−1.05)

^1^ All dimensions are in mm.

**Table 11:** Microstrip antenna geometrical parameters obtained in Phase 1 of Experiment 2.

Parameter	Value ^1^
R	14.1633
L1	16
S2	2.4353
dp	3.5
Wp	4.5
Ls	0.554
Lt	18.3385
dl	−1.5

^1^ All dimensions are in mm.

**Table 12:** Microstrip antenna geometry obtained in Phase 2 of Experiment 2.

Parameter	R	Lt	L1	S2	dl	dp	Wp	ls
Value ^1^	14.3590	18.5644	16.3893	3.1039	−1.7826	2.7071	5.7709	0.3966

^1^ All geometrical parameters are in mm.

**Table 13:** Statistics of bandwidth expansion of Experiment 2.

Parameter	Original Bandwidth (GHz)	Bandwidth Extension of Phase 1 (GHz)	Bandwidth Extension of Phase 2 (GHz)	Total Bandwidth Extension (GHz)	Total Bandwidth Extension %
$S_{11}$_low	0.4	0	0	0	0
$S_{11}$_high	1.26	0.26	0.08	0.34	27%
AR_low	0.14	0.02	0.02	0.04	28%
AR_high	0.04	0.04	0.06	0.1	250%

**Table 14:** Statistics on the increase in bandwidth for different algorithms in Experiment 2.

Parameter	ICOA–TD3 ^1^ (GHz)	GA (GHz)	PSO (GHz)	DE (GHz)	BO (GHz)
$S_{11}$_low	**0**	−1.8	−0.22	−0.22	−0.18
$S_{11}$_high	**0.34**	−0.22	0.26	0.32	−0.06
AR_low	**0.04**	**0.04**	0.02	**0.04**	0.02
AR_high	0.1	0.22	0.38	0.4	**0.42**

^1^ Its value is the data in column 5 (Total Bandwidth Extension) of Table 13.

## Data Availability

The data presented in this study are available on request from the corresponding author.

## Abbreviations

The following abbreviations are used in this manuscript:

ICOA	improved crayfish optimization algorithm
COA	crayfish optimization algorithm
TD3	twin delayed deep deterministic policy gradient
$S_{11}$	reflection coefficient
AR	axial ratio
PSO	particle swarm optimization
NSGA-II	non-dominated sorting genetic algorithm
DoE	differential evolution
ABC	artificial bee colony
BPSO	binary particle swarm optimization
GWO	gray wolf optimizer
ANN	artificial neural network
LSTM	long short-term memory
DNN	deep neural network
MLHS	maximin latin hypercube sampling
